# Role of zinc in severe pneumonia: a randomized double bind placebo controlled study

**DOI:** 10.1186/1824-7288-38-36

**Published:** 2012-08-02

**Authors:** Gauri S Shah, Ashok K Dutta, Dheeraj Shah, Om P Mishra

**Affiliations:** 1 Department of Pediatrics, B.P. Koirala Institute of Health Sciences, Dharan, Nepal; 2 Department of Pediatrics, Lady Harding Medical College & Kalawati Saran Children Hospital, New Delhi, India; 3 Department of Pediatrics, University College of Medical Sciences & GTB Hospital, Delhi, India; 4 Department of Pediatrics, Institute of Medical Sciences, Banaras Hindu University, Varanasi, India

**Keywords:** Pneumonia, Children, Zinc

## Abstract

**Background:**

Pneumonia is a leading cause of morbidity and mortality in children.

**Objective:**

The aim of study was to evaluate the efficacy of Zinc supplementation in treatment of severe pneumonia in hospitalized children.

**Design/Methods:**

A double blind randomized, placebo- controlled clinical trial conducted at a tertiary care centre of a teaching hospital. Children with diagnosis of severe pneumonia were randomly assigned to receive supplementation with either elemental zinc or placebo by mouth at the time of enrollment. From day 2, they received 10 mg of their assigned treatment by mouth twice a day for 7 days along with standard antimicrobial therapy.

**Results:**

The baseline characteristics like age, sex, weight, weight Z score, height, height Z score, weight for height Z score and hemoglobin were comparable in both study groups. The respiratory rate, chest indrawing, cyanosis, stridor, nasal flaring, wheeze and fever in both groups recorded at enrollment and parameters did not differ significantly between the two groups. The outcome measures like time taken for resolution of severe pneumonia, pneumonia, duration of hospital stay, nil per oral, intravenous fluid, oxygen use, treatment requiring 2^nd^ line of drug and 3^rd^ line drug were evaluated and found to be same.

**Conclusion:**

The present study did not show a statistically significant reduction in duration of severe pneumonia, or reduction in hospital stay for children given daily zinc supplementation along with standard antimicrobial therapy. Therefore, zinc supplementation given during the acute episode does not help in short term clinical recovery from severe pneumonia.

## Introduction

Worldwide, pneumonia is the leading cause of pediatric morbidity and mortality. It is estimated that pneumonia is responsible for >2 million deaths each year in children below 5 years of age, and contributes 20% of the annual deaths in this age group [1]. Approximately 95% of the pneumonia-related deaths occur in developing countries, and the younger age groups have the highest risk of death. Zinc supplementation lowers the risk of acute respiratory illnesses and diarrhea in children [2]. A recent clinical trial conducted in Bangladesh suggested that zinc supplementation given with empiric antimicrobial therapy can significantly shorten the duration of severe pneumonia, tachypnea, hypoxia, and chest indrawing and duration of hospital stay for young children with pneumonia [3]. However, therapeutic trials of Zn conducted in Indian hospitalized children with severe pneumonia demonstrated no overall effect [4,5]. Valentiner-Branth et al. [6] demonstrated that Zn neither reduced the risk of treatment failure nor hasten the recovery of non-severe or severe pneumonia in Nepalese children between 2–35 month of age. Many children in Nepal, especially below 5 years of age, are admitted with severe pneumonia in pediatric wards requiring antimicrobial therapy with prolonged hospital stay. In view of previous studies, which have evaluated the role of zinc in children with pneumonia either below 2 or 3 years of age with variable reports [3,6]. The present study was undertaken to find out the efficacy of zinc supplementation in the treatment of severe pneumonia, beside standard antimicrobial therapy, in hospitalized Nepalese children, aged 2 months to 5 years, by comparing the different outcome measures between zinc supplemented and placebo groups.

## Patients and methods

### Study design and setting

This study was a randomized, double-blind, placebo-controlled clinical trial conducted between June 2008 to August 2009 at Department of Pediatrics, B. P. Koirala Institute of Health Sciences, Dharan, Nepal. This trial was registered in the CTRI Website and CTRI No. is: CTRI/2008/091/00006.

Children between 2 months to 5 year of age admitted to the pediatric wards were assessed and considered eligible for enrollment in the trial if they fulfilled the criteria for a diagnosis of severe pneumonia. Severe pneumonia was defined as per WHO/IMCI (Integrated Management of Childhood illness) guide line [7] and whose parents provided written informed consent were included in the study. Patients who had congenital cardiac or renal disease, severe malnutrition (as defined by WHO), concomitant diarrhea, severe anemia (Hemoglobin <8 g/dl), complicated pneumonia, congenital lung anomalies, chronic cough, documented tuberculosis, recurrent wheezing and children who were receiving zinc supplements were excluded from the study. Written consent was obtained after the parents or guardians read study information. If the parents or guardians were illiterate, the content of the written consent was read to them, and consent was documented by a thumbprint impression of one of the parents or guardians in the presence of an unrelated witness. The study was approved by the Institute Ethical Committee and project review board of B. P. Koirala Institute of Health Sciences. A detailed history, physical and systemic examinations were carried out and recorded in a predesigned data sheet at the time of enrollment. The randomization codes were generated by the Deurali Janta Pharmaceutical, Nepal who provided the coded medicine strips for study. The codes were broken only at the end of study.

### Intervention

We calculated the sample size using EPI Info (WHO recommended software for sample size) with an effect size of 20%, 5% chance of type I and 10% chance of type II error, keeping Odds ratio 3 times more benefit in supplemented group. This calculation resulted in a sample size of 55 in each group. Children with a diagnosis of severe pneumonia were randomly assigned to receive supplementation with either elemental zinc or placebo tablets. Each tablet contained 10 mg zinc sulfate or 10 mg placebo. The zinc and placebo tablets were identical in appearance, consistency and taste. The supplements were formulated and manufactured as dispersible tablets by Deurali Janata Pharmaceuticals, Nepal and packaged in a strip of 10 tablets. The back of strip was labeled with identification number. Children received 20 mg of either zinc sulfate or placebo by mouth at the time of enrollment. From day 2, they received 10 mg of their assigned treatment by mouth twice a day for total of 7 days. All enrolled children were treated according to the standard protocol for treatment of severe pneumonia. The primary outcome measures were decrease in duration of severe pneumonia and pneumonia and secondary outcome measures were decrease in duration of nil per orally, intravenous fluid, use of oxygen and treatment failures requiring 2^nd^ and 3^rd^ line antibiotics treatment as defined in treatment protocol. They were treated parentally with a combination of cefotaxime and gentamicin if the age of child was less then 1 year and with cefotaxime alone when the age of child was more than 1 year. Patients who failed to improve within 48 hr on this regimen, cloxacillin was added. If not improved within next 48 hr with cloxacillin, then it was replaced by vancomycin. If any suspicion about Chlamydia or Mycoplasma was there, then azithromycin was added.

### Follow up during hospital stay

During hospitalization, the child’s condition was assessed 8 hourly. Respiratory rate was measured for a full one min. The count was done at a time when the child was not crying. Pulse oximetry was measured with the use of a probe (Nellcor Inc, Hayward, CA) placed on a finger or toe. Axillary temperature was measured by using a standard mercury thermometer. The presence of cough, crepitations, wheezing, chest indrawing, cyanosis, inability to feed, and lethargy/unconsciousness were also noted. Enteral feeds were started once the child’s respiratory distress improved and oxygen saturation was ≥93%, and the baby was able to tolerate small sips of feeds. Feeds were started as early as possible to optimally balance fluid and caloric intake. Oral antibiotic cefpodoxime proxetil was started when the child was feeding well and when oxygen saturation and respiratory rate were stabilized. Once the patient was on oral antibiotic, he or she remained under observation in the ward for a further 24 hr before discharge. Study subjects were discharged when they were being fed entirely with oral feeds, the respiratory rate was normalized, oxygen saturation was ≥93%, and the attending pediatrician decided that the patient’s clinical condition had resolved and did not require further hospital stay.

### Statistical analysis

The data was analyzed by using SPSS Version 12.0 software. The Student’s *t* test was used of data showing normal distribution and Mann Whitney *U* test for non-Gaussian distribution. Chi-square and Z tests were used for comparisons of proportions as appropriate, to assess the treatment group differences. A p value <0.05 was considered as statistically significant.

## Results

**Figure 1:**
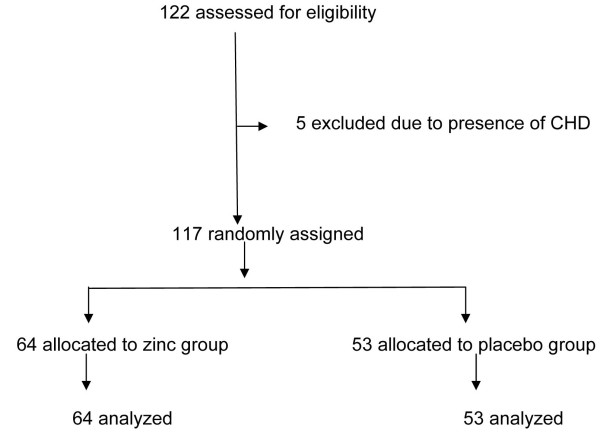
Study flow.

Between June 2008 and August 2009, 122 children who were eligible for participation in the trial (Figure 1); five children were excluded from the study as because they did not meet the inclusion criteria due to detection of congenital heart disease. The remaining 117 children were enrolled, and 64 were randomly assigned to take Zinc tablet and 53 were assigned to take placebo which was not known to us as it was randomized, double blind controlled trial. Baseline characteristics of the children between placebo and zinc treatment groups like age, sex, weight, weight Z score, height, height Z score, weight for height Z score and hemoglobin were comparable (Table 1). The clinical sign and symptoms like respiratory rate, chest indrawing, stridor, cyanosis, nasal flaring, wheeze and fever were evaluated and no significant differences were found between zinc and placebo groups and observations are shown in Table 2.

**Table 1:** Baseline characteristics in zinc and placebo group

**Characteristics**	**Zinc group n = 64**	**Placebo group n = 53**	**P- value**
Age (years)	9 (5.0,14.7)	10 (6.0,18.5)	0.116*
Gender n (%) Male Female	43 (67% 21 (32.8%)	33 (62.3%) 20 (37.7%)	0.578**
Weight (Kg)	7.92 +2.08	8.27 +2.13	0.382***
Weight Z score	−1.05(−1.577, 0.040)	−1.25 (−2.28, −0.180)	0.074*
Height (cm)	70.98 +8.45	73.37 +11.42	0.196***
Height Z score	−875(−2.12, 0.385)	−1.38(−2.72, 0.70)	0.249*
Weight-for-height Z score	−0.510(−1.41, 0.830)	−0.770(−1.77, 0.275)	0.241*
Hemoglobin (g/dl)	10.6 +1.3	10.4 +1.1	0.431***

Data are expressed as median, mean + SD, Figures in parentheses indicate interquartile range.

*Mann Whitney *U* test, ** Z test, *** Student’s *t* test.

**Table 2:** Clinical signs and symptoms in zinc and placebo groups

**Characteristics**	**Zinc n = 64**	**Placebo n = 53**	**P- value**
Respiratory rate at enrolment Mean (SD)	64 (8)	63 (11)	0.753*
Chest indrawing n (%)	64 (100%)	53 (100%)	---
Stridor n (%)	6 (9.3%)	3 (5.6%)	0.453**
Cyanosis n (%)	17 (26.6%)	9 (16.9%)	0.215**
Nasal flaring n (%)	22 (34.4%)	25 (47.2%)	0.160**
Wheeze n (%)	43 (67.2%)	40 (74.5%)	0.326**
Fever n (%)	64 (100%)	53 (100%)	---

n – Number of cases, *Student’s *t* test, **Z test.

The outcome measures between the two groups like median duration of severe pneumonia, pneumonia, hospitalization, nil per orally, intravenous fluid, oxygen use, treatment requiring 2^nd^ line and 3^rd^ line drugs were compared. It was observed that none of the parameters were statistically significant between zinc and placebo groups (Table 3).

**Table 3:** Comparison of Outcomes in Zinc and Placebo Groups

**Outcome**	**Zinc n = 64**	**Placebo n =53**	**P**
Duration of hospitalization (hours)	73.5 (49.5,107.5)	72 (48.0,87.7)	0.193*
Duration of severe pneumonia (hours)	34.2 (21.0,48.0)	26 (16.0,46.0)	0.219*
Duration of pneumonia (hours)	40 (24,54.2)	43 (18.5,72.0)	0.943*
Duration of nil-per-orally (hours)	10 (0.0,23)	12 (0.0,18)	0.771*
Duration of intravenous fluid (hours)	22.0 (12.0,31.0)	16 (1.5,32.5)	0.258*
Duration of oxygen use (hours)	10 (0.0,22.0)	11.0 (0.0,23.5)	0.684*
Treatment failure requiring 2^nd^ line drugs	14 (21.9%)	15 (28.3%)	0.423**
Treatment failure requiring 3^rd^ line drugs	1 (1.6%)	1 (1.9%)	0.893**

n - Numer of cases, Data are expressed as median, Figures in parentheses indicate interquartile range and percentage.

*Mann Whitney *U* test, ** Z test.

## Discussion

Zinc plays an important role in the development and maintenance of host defense against infections [8,9]. The therapeutic benefit of oral zinc in diarrhea has been well documented in a Cochrane review of 18 trials which showed that it shortened the recovery time in children with acute or persistent diarrhea in the age group of 6 months to 5 years [10] Further, it has also been shown that routine zinc supplementation lowers the risk of acute respiratory infections and clinical pneumonia in children [2].

Keeping these considerations, the present study was carried out to find out the influence of co-administration of zinc with standard antimicrobial therapy in a double- blind randomized controlled clinical trial with primary outcome measures as reduction in duration of severe pneumonia and pneumonia. Overall basic characteristics and clinical signs and symptoms were comparable between zinc and placebo groups. We did not find any statistically significant median reduction in duration of severe pneumonia, pneumonia, hospital stay, nil per orally, intravenous fluid, duration of oxygen use, treatment requiring 2^nd^ line or 3^rd^ line antibiotic drugs in zinc supplemented in comparison to placebo group along with standard antimicrobial therapy. Other studies [4,5] also did not find any positive benefit of reduction in duration of severe pneumonia or pneumonia in zinc supplemented group. Recently, a study from our country also demonstrated that adjuvant zinc neither reduced the risk of treatment failure nor hasten the recovery from non- severe or severe pneumonia in Nepalese children in the age group of 2–35 months of age [6]. In contrast, results from a trial in Bangladesh suggested that zinc supplementation given with empiric antimicrobial therapy significantly shortened the duration of severe pneumonia, tachypnoe, hypoxia, and chest indrawing and the hospital stay for young children with pneumonia. The difference in outcome in different trials could be better explained if pre and post- treatment plasma zinc levels estimation would have been done. As such benefit is expected if children are in zinc deficient state. In our study, concomitant severe malnutrition cases were excluded, which often have co-existing zinc deficiency. Therefore, it may be possible that study population was not zinc deficient and benefit of zinc therapy was not found. However, it is a matter of conjecture and difficult to conclude as plasma level of zinc was not estimated. We could identify no sub-group that benefited from zinc supplementation especially when the data was analyzed in relation to those cases t had wheezing also. Similar was the observation reported by Brooks et al. also in their study [3].

## Conclusion

This study did not show a statistically significant reduction in duration of severe pneumonia, and duration of pneumonia as primary outcome measures. The duration of hospitalization, nil per orally, oxygen use and treatment failure were comparable between zinc and placebo group. Thus, it appears that zinc supplementation given during acute episode does not help in short- term clinical recovery from severe pneumonia in non- malnourished children.

### What is already known on this topic

Zinc supplementation in treatment of severe pneumonia in children, aged 2 to 35 months, demonstrated inconsistent results.

### What this study adds

Zinc therapy does not shorten the duration of severe pneumonia or pneumonia in hospitalized children between 2 months to 5 years of age.

## Competing interests

The authors declare that they have no competing interests.

## Authors’ contributions

GSS & DS- Designed, conducted and analyzed the study, AKD- Designed and conducted and OPM-Analyzed the data and drafted the manuscript.

## Funding

The study was funded by research grant provided by B. P. Koirala Institute of Health Sciences, Dharan, Nepal.
